# Telemedicine and Cultural Competency in Dementia Care: Mapping Stakeholder Roles in Digital Toolkit Development in SSA: A Systematic Review

**DOI:** 10.1111/hex.70489

**Published:** 2025-11-16

**Authors:** Abiodun Adedeji, Huseyin Dogan, Festus Adedoyin, Michelle Heward

**Affiliations:** ^1^ Department of Computing and Informatics, Faculty of Science and Technology Bournemouth University Bournemouth UK; ^2^ Department of Psychology, Faculty of Science and Technology Bournemouth University Bournemouth UK

**Keywords:** cultural competency, dementia, digital toolkit, stakeholder engagement, Sub‐Saharan Africa (SSA), telemedicine

## Abstract

**Background:**

Dementia is a growing concern in Sub‐Saharan Africa (SSA), particularly Nigeria, where care is hindered by weak infrastructure, stigma, and limited culturally responsive services. Telemedicine offers promise but faces adoption challenges in low‐resource settings.

**Objective:**

To evaluate digital interventions for dementia care in SSA and examine how culturally tailored telemedicine is shaped by stakeholder input, it may reduce caregiver burden and improve care delivery.

**Methods:**

Following PRISMA guidelines, 1650 records were screened: across PubMed, Scopus, PsycINFO, and Google Scholar; 20 studies met inclusion criteria. Thematic analysis identified trends across intervention types, populations, and barriers.

**Results:**

Five core themes emerged: barriers to care (30%), telemedicine opportunities (25%), stakeholder involvement (23%), cultural influences (20%) and technological feasibility (17%). Mobile health tools showed potential when culturally adapted. However, poor infrastructure, digital literacy and traditional beliefs constrained adoption. Engaging caregivers, clinicians and community leaders improved solution relevance and uptake.

**Conclusions:**

Culturally informed, co‐designed telemedicine models can strengthen dementia care delivery in SSA, stakeholder‐driven digital models may serve as a scalable blueprint for other low‐resource contexts.

**Patient or Public Contribution:**

This review incorporated studies that engaged patients, informal caregivers, clinicians, policymakers and community leaders in the codesign and implementation of digital dementia care tools. Their lived experiences and cultural insights informed the development of interventions that are not only technologically feasible but also socially and “culturally appropriate”.

## 1 Introduction

Dementia is a rising public health concern in Sub‐Saharan Africa (SSA), where health systems are under‐resourced, and awareness remains low [1]. Cultural beliefs often frame cognitive decline as a natural aspect of aging or a supernatural condition, contributing to late diagnosis and limited access to care, particularly in rural areas [2, 3]. These delays raise family burden, as informal caregivers manage most care under difficult conditions [4].

In many regions, stigma, lack of trained specialists and weak diagnostic infrastructure deepen the treatment gap [3, 5, 6].

Telemedicine offers a promising solution to bridge gaps in dementia care by enabling the remote delivery of services tailored to local languages and cultural practices, thus addressing both distance and cost barriers [7, 8]. The growing penetration of smartphones across SSA further enhances the feasibility of mobile health (mHealth) interventions in the region [9, 10].

Cultural competency refers to the ability to effectively deliver healthcare services that meet the social, cultural and linguistic needs of patients. It is related but distinct from cultural sensitivity (awareness of cultural differences), cultural humility (lifelong self‐reflection) and cultural safety (addressing power imbalances) [11, 12]. This review aims to map stakeholder roles and cultural considerations in telemedicine for dementia care across SSA.

### 1.1 Conceptual Framing

Our interpretation draws on the technology acceptance model to consider perceived usefulness and ease of use for telemedicine [13]. The Campinha‐Bacote process of cultural competence to guide culturally responsive care and where relevant [14], the consolidated framework for implementation research (CFIR) [12] and stakeholder theory to interpret context and roles. These frameworks are referenced throughout to support theoretical continuity from methods to interpretation [15].

### 1.2 Research Questions

This study explored how telemedicine is used to support dementia care in SSA, with a focus on cultural relevance and caregiver support, consequently, this systematic review investigates the following pertinent questions: What telemedicine tools are used in dementia care across SSA? How do these tools support caregivers and improve care quality? How do cultural beliefs affect dementia care practices? What roles do key stakeholders play in telemedicine design and delivery? What barriers limit the adoption of culturally appropriate telemedicine systems?

#### 1.2.1 Current Landscape of Dementia in SSA

Dementia is a rising public health challenge globally, with potential ramifications in low‐and‐middle‐income‐countries (LMICs), such as those in SSA where demographic transitions, population aging and underdeveloped health systems are associated with a rising care burden [1, 3]. In SSA, over 3 million people are projected to live with dementia by 2030, while globally an estimated 55 million people currently live with dementia, rising to 78 million by 2030 and 139 million by 2050 [1, 16].

Cultural interpretation often delay diagnosis, as dementia symptoms are sometimes misattributed to normal aging or to spiritual and supernatural causes [17, 18]. These beliefs are deeply embedded in broader socio‐cultural contexts that warrant respectful engagement rather than dismissal [19]. Health systems in the region continue to prioritise infectious diseases, with limited attention given to non‐communicable conditions like dementia [20, 21]. Diagnosis tools and public health strategies are often lacking or inadequate [1]. Additionally, comorbidities such as hypertension and diabetes contribute significantly to dementia prevalence, particularly in rural areas with poor health service coverage [22].

### 1.3 Telemedicine as an Emerging Solution

Telemedicine holds a significant promise for dementia care, particularly given that mobile phone use exceeds 80% in some areas of SSA [23, 24, 25]. Digital tools support remote assessments and caregiver education [26, 27]. In Tanzania, cognitive screening apps designed with local languages and customs in mind have shown high accuracy and acceptance among users [28]. However, the significant barriers remaining only 29% of SSA has reliable internet access, and digital literacy levels are low [29]. Addressing these disparities is essential for scaling telemedicine effectively and equitably. Table 1 summarises regional comparisons of dementia care characteristics including prevalence, key challenges and current strategies across SSA, Asia, Latin America and Japan, drawing on data from multiple studies [1, 28].

**Table 1:** Regional comparison of dementia care characteristics.

Region	Prevalence of dementia	Key challenges	Current strategies
SSA	12%–22% among those aged 60+; projected to exceed 3 million by 2030	Insufficient healthcare funding	Collaborations with international health organisations
		Shortage of specialised professionals	Initiatives like the African Dementia Consortium
		Stigma and cultural beliefs	
		Limited diagnostic tools	
Asia	Varies significantly by country, high informal care reliance	Cultural stigma around mental health	Awareness campaigns
		Lack of awareness	Training for informal caregivers
		Underprepared healthcare systems	
Latin America	Varies; compounded by economic disparities	Economic inequalities	Integrated care within general NCD management
		Inadequate financing	Community outreach programmes
		Structural healthcare issues	
Japan	Increasing due to aging population	Cultural resistance to institutional care	Telemedicine solutions for remote assessments
		Burden on families	Family training programmes
		Stigma around dementia	

*Note:* Summary of dementia prevalence, barriers and healthcare responses across high‐income countries, low‐and middle‐income‐countries (LMICs), and SSA.

### 1.4 Stakeholder Roles in Nigeria's Dementia Care Framework

Mapping the roles and interests of stakeholders across the health system improves alignment and adoption of digital dementia care solutions [30, 31]. Nigeria's toolkit project demonstrated that early stakeholder engagement supports sustainability by facilitating the identification of potential challenges and co‐creating user‐friendly tools [32]. Table 2 presents the role of key stakeholders in Nigeria's dementia care framework, highlighting their unique contributions to the implementation of telemedicine and other digital health solutions. Engaging these actors meaningfully is critical to achieving sustainable, culturally appropriate interventions.

**Table 2:** Role of stakeholders in Nigeria's dementia framework.

Stakeholders	Contributions
Healthcare providers	Clinical guidance
Families and caregivers	Practical insights
Policymakers	Resource allocation
Tech experts	Toolkit development
Researchers	Innovation
NGOs and community	Outreach and support
Private sector	Funding and expertise
International networks	Knowledge exchange

*Note:* Illustrates the contributions of healthcare providers, caregivers, policymakers, tech experts, NGOs and others in dementia care in Nigeria.

**Figure 1:**
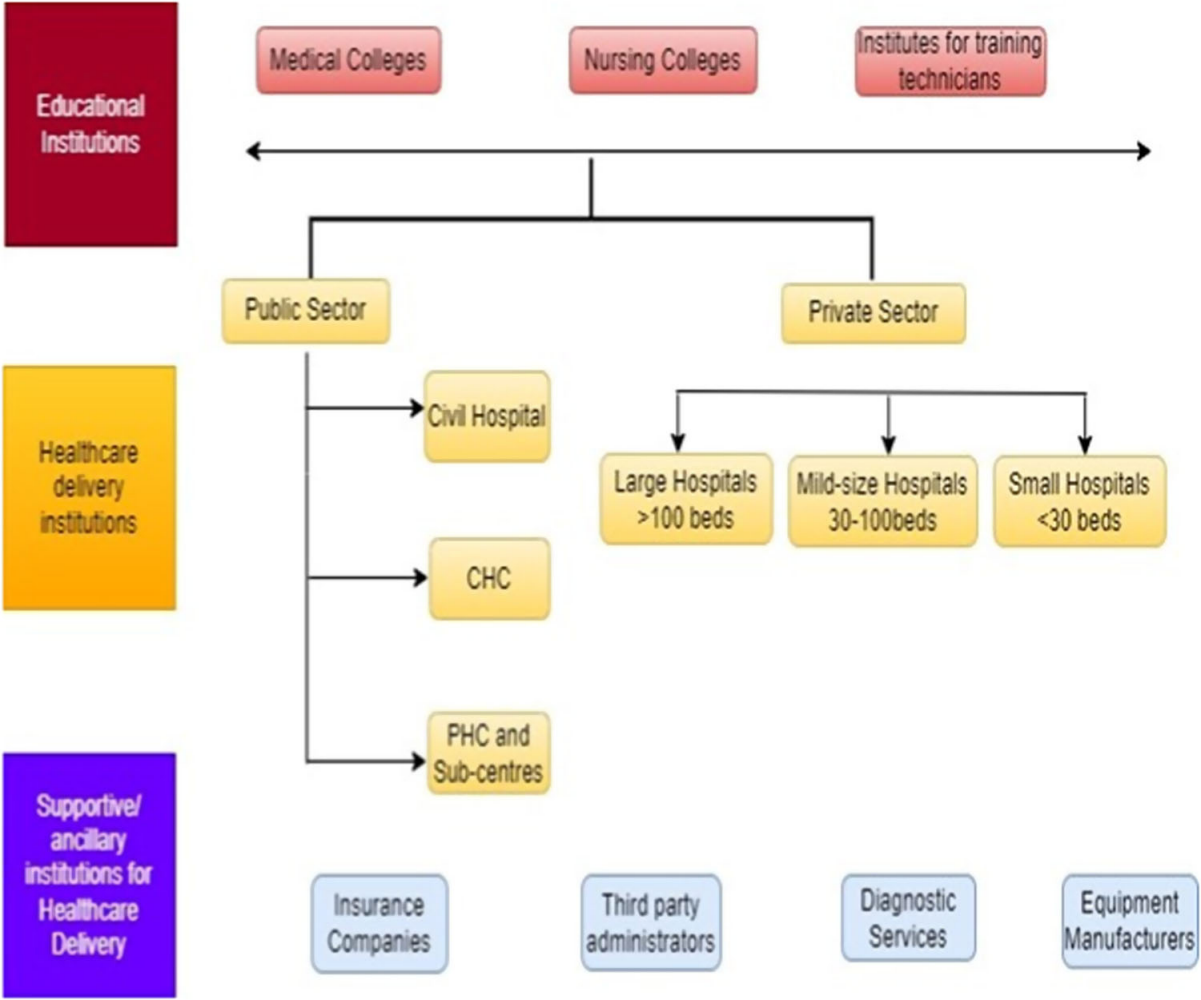
Stakeholder mapping in Nigeria's dementia care framework. The figure shows the interconnected roles of healthcare professionals, families and caregivers, policymakers, technology experts, researchers, NGOs and community organisations, the private sector (including private hospitals and telehealth providers) and international networks. MoH, Ministry of Health; NGO, nongovernmental organisation; PHC, primary health care.

Caregivers offer critical insights into daily challenges of dementia care and intervention usability (see Table 2), while collaboration with developers ensures that designs are accessible, secure and culturally appropriate. Beyond home and clinic, NGOs, advocacy groups and gas roots organisations provide outreach and support, reinforcing the importance of engaging every stakeholder meaningfully to ensure successful and sustained adoption of digital solutions [33]. The mapping of stakeholder roles is presented in Figure 1, illustrating their interconnected contributions to dementia care in Nigeria (see Figure 1).

## 2 Methodology

This review set out to explore how telemedicine might help improve culturally sensitive dementia care in SSA, especially in countries with limited resources like Nigeria. To do this, it brought together real‐world findings and perspectives from key stakeholders, drawing from two related areas of research: one looking at how telemedicine is being used across the region, and the other focused on creating practical, community‐based tools for dementia care in Nigeria.

### 2.1 Search Strategy and Data Collection

This systematic review was conducted following the PRISMA 2020 guidelines [34]. However, the review protocol was not registered in PROSPERO or any other protocol registry due to the exploratory nature and time constraints of the project.

Databases searched included PubMed, Scopus, Web of Science, CINAHL and Google Scholar for relevant studies. The search was conducted in November, 2024 and included all articles published up to that date. The full search strategy used for Scopus is provided in Appendix A.

The initial database search identified 1650 records; after removing 200 duplicate records, 1450 titles and abstracts were screened for relevance. Of these, 1320 records were excluded due to low contextual relevance, leaving 100 full‐text reports sought for retrieval from the database search. In parallel, citation searching identified an additional 20 records, which were also sought for retrieval. All 120 reports (100 from database and 20 from citation searching) were retrieved in full text and assessed for eligibility. During full text review, 80 reports from the database search and 20 from citation searching were excluded, with documented reasons, leaving 20 studies included in the final review.

Of the 20 included studies:

- Twelve were empirical or implementation studies exploring telemedicine and dementia care in SSA, with examples from Tanzania, Ghana, Kenya, Nigeria and South Africa.
- Eight focused on participatory design, stakeholder engagement, and the development of digital health toolkits in Nigeria.

Together, these studies offer a well‐rounded look at how telemedicine can be both practical and culturally relevant for dementia care in the region.

### 2.2 Inclusion and Exclusion Criteria

Studies were included if they reported on telemedicine or digital health interventions in dementia care, were conducted in SSA, and addressed cultural competency, barriers, facilitators, stakeholder engagement or outcomes. Studies were excluded if they focused solely on pharmacological treatments, were not related to dementia or telemedicine or lacked relevance to cultural or contextual factors.

### 2.3 Data Extraction and Synthesis

For each included study, key data were extracted and organised according to the country or region of focus, study population and setting, type of telemedicine intervention, cultural competency strategies, barriers and facilitators to adoption, stakeholder involvement and reported outcomes. Data extraction was performed independently by two reviewers H.D. and F.A. to minimise bias. Any discrepancies were discussed and resolved by consensus, if consensus could not be achieved, a third reviewer was conducted to adjudicate.

Thematic synthesis was used to identify and organise common themes across the included studies, following the approach described by Wakawa et al. [35].

#### 2.3.1 Stakeholder Mapping Integration

This review highlights stakeholder mapping in Nigeria's codesign of a digital dementia care toolkit, emphasising the value of involving healthcare professionals, caregivers, policymakers, tech‐experts and advocacy groups in shaping innovation [36, 37, 38]. Through stakeholder mapping, the review was able to take a closer look at key factors such as:

- The cultural adaptability of digital health tools
- Community buy‐in and usability
- The integration of traditional healthcare beliefs into formal dementia care
- Data privacy, design requirements and training needs

## 3 Results

### 3.1 Study Selection

**Figure 2:**
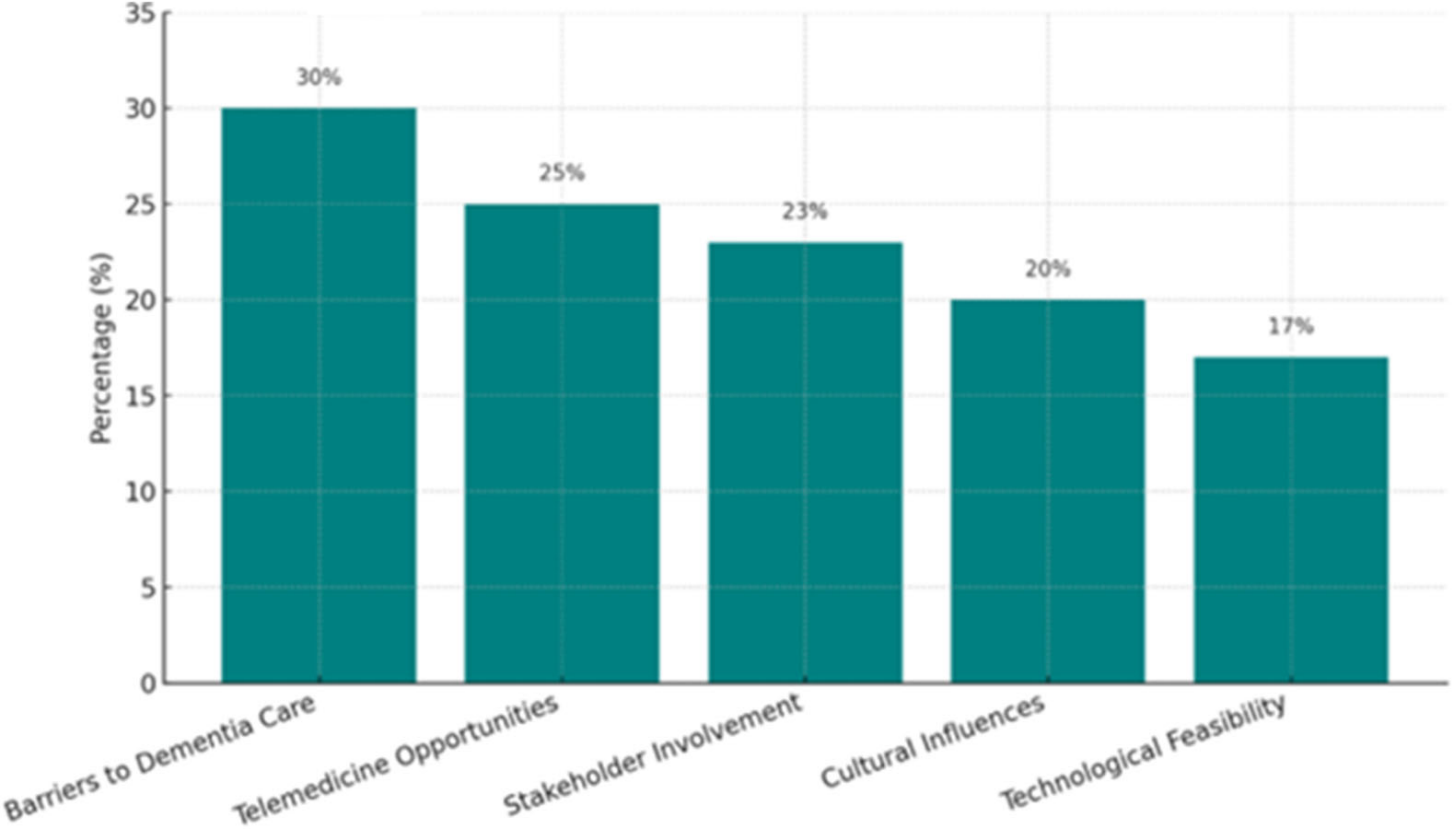
Core themes in the digitisation of dementia care include barriers to care, telemedicine opportunities, cultural influences, stakeholder involvement and technological feasibility [25, 38, 47, 51]. (Barriers to care, telemedicine opportunities, cultural influences, stakeholder involvement, and technological feasibility).

A total of 1650 records were identified through database searches, and an additional 20 records were identified through citation searching. After removing 200 duplicates, 1450 titles and abstracts were screened for relevance, with 1320 excluded due to low contextual relevance. Subsequently, 120 full‐text articles (100 from database searches and 20 from citation searching) were retrieved and assessed for eligibility. Of these, 100 were excluded (80 from the database search and 20 from citation search) with documented reasons, resulting in 20 studies included in the final review (Figure 2).

**Figure 3:**
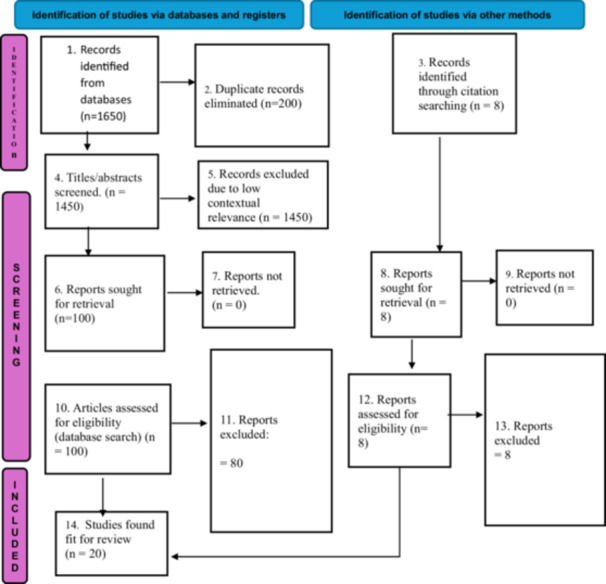
Prisma 2020 flow diagram illustrating the selection process of studies included in the review. (Depicts the flow of studies through the identification, screening, eligibility and inclusion phases of the review).

The study selection process is illustrated in Figure 3, and the methodological quality of included studies is summarised in Table 3.

**Table 3:** Methodological quality of included studies.

Study	Design	Appraisal tool	Quality rating
[28]	Qualitative	CASP	High
[36]	Mixed methods	JBI	Moderate
[37]	Qualitative	CASP	High
[38]	Participatory	CASP	Moderate
[39]	Observational	JBI	Moderate
[40]	Narrative review	CASP	Moderate
[41]	Overview	JBI	Low
[42]	Exploratory	CASP	Moderate
[13]	Phenomenological	CASP	High
[27]	Review	JBI	Moderate
[28]	Case study	CASP	Low
[43]	Qualitative	CASP	High
[44]	Cross‐sectional	CASP	Moderate
[45]	Quantitative	JBI	High
[46]	Scoping review	JBI	Moderate
[47]	Expert commentary	N/A	N/A
[48]	Narrative review	CASP	Moderate
[37]	Participatory design	CASP	High
[38]	Tech development	JBI	Moderate
[14]	Policy analysis	CASP	Moderate
[36]	Qualitative	CASP	High

*Note:* Details the design, appraisal tool and quality.

### 3.2 Characteristics of Included Studies

A total of 20 studies were included in this review, reflecting a diverse body of dementia care research and innovation across SSA, with Nigeria serving as the focal point. Nine studies were conducted in Nigeria [36, 37, 38, 39, 40, 45, 49]. Followed by region‐wide SSA studies (*n* = 3) [1, 37, 50], Africa‐focused studies (*n* = 3) [1, 41, 51], Ghana (*n* = 2) [40, 44], Tanzania (*n* = 1) [28], South Africa (*n* = 1) [51], and one from the United States focusing on remote engagement applicable to SSA contexts [43].

The studies employed a range of methodological approaches: Qualitative designs were the most common (*n* = 12), followed by observational studies (*n* = 5) and participatory or codesign approaches (*n* = 3). Interventions examined included telehealth consultations, app‐based cognitive screening, digital toolkits, strategic planning, stakeholder engagement and reviews of dementia care models.

Stakeholders engaged in these studies included caregivers (*n* = 14), healthcare professionals (*n* = 10), policymakers (*n* = 6), NGOs/community‐based organisations (*n* = 4) and technology developers (*n* = 5). Caregivers were most frequently represented, emphasising their central role in dementia care in SSA. Table 4 summarises the key characteristics of the included studies, including authors, country or region, study population, type of intervention and main findings.

**Table 4:** Characteristics of included studies.

Author(s)/Year	Country/Region	Study/Population	Intervention	Key findings
[41]	Africa	General African population	Remote consultants, specialist care	Telemedicine improves access but faces infrastructure/cultural barriers
[1]	Africa	Older adults in low‐and‐middle‐income‐country (LMICs)	Not focused on telemedicine	Prevalence varies research and care gaps exist
[52]	Nigeria	Patients and providers	Not applicable	Cultural beliefs delay diagnosis in Nigeria
[40]	Ghana	Ghanaian population	Telemedicine during COVID‐19	COVID‐19 increased the use of telehealth; more support needed
[28]	Tanzania	Adults aged less than or equal to	App‐based cognitive screening	Apps have good sensitivity and culturally relevant tools help access
[44]	Ghana	School students	None	High stigma; need for culturally appropriate education
[32]	Sub‐Sharan Africa	Multiple SSA populations	None	Age and education linked to dementia risk
[18]	Nigeria	Public health facilities	e‐health record systems	Most facilities use paper records, poor data infrastructure
[31]	SSA	Population affected by stroke	Conference proposal	Need for SSA‐specific interventions and education
[46]	Africa	Patients with dementia	Telehealth/precision medicine	Telemedicine aids personalisation, but access barriers remain
[45]	Nigeria	Patients 60+ with dementia	Telehealth follow‐up support	High Alzheimer's rate: poor follow‐up due to stigma
[49]	Nigeria	Not specified	Strategic planning and remote care	No national dementia plan; calls for integrated care
[38]	Nigeria	Tech development and caregivers	Digital toolkit development	Stakeholder‐centred design enhances usability
[37]	Nigeria	Health professionals and caregivers	Participatory codesign	Involving caregivers increases toolkit adoption
[36]	Nigeria	Health stakeholders	Stakeholder consultation	Mapping improves engagement and sustainability
[49]	South Africa	Family caregivers	None	Cultural roles shape care experience
[43]	USA	People with dementia	Remote engagement	Caregivers improve research insights

*Note:* Summarises author/year, study design, population, interventions, key findings and rating of each included study.

### 3.3 Comparative Insights Across SSA

Comparative insights across SSA. The Nigeria studies emphasised participatory codesign and toolkit development, with early engagement of caregivers and clinicians to improve cultural fit and usability [36, 37, 38, 49]. In Ghana, telehealth use accelerated during COVID‐19, but sustained adoption was limited by infrastructure and stigma [40, 44]. In Tanzania, app‐based cognitive screening showed good feasibility when adapted to local languages and norms [28]. In South Africa, studies during and after COVID‐19 highlighted the promise of digital follow‐up but reinforced persistent access and literacy gaps [51]. Across cross‐SSA syntheses, the main constraints connectivity, device availability and digital skills were consistent, while locally tailored content and stakeholder buy‐in were key enablers [9, 10, 29, 41].

### 3.4 Thematic Findings

The reported percentages for thematic findings (e.g., barriers 30%, opportunities 25%) were derived from the proportion of studies addressing each theme out of the total studies, following a qualitative content analysis approach [53].

Five key themes emerged from the synthesis, illustrating the challenges and opportunities for telemedicine in culturally competent dementia care in SSA (Figure 2).

1. Barriers to dementia care: Shortages of specialists, inadequate tools, underfunding and stigma delayed care [42, 48, 54].
2. Opportunities for telemedicine: Remote tools and virtual consultations increased access when culturally adapted, despite poor infrastructure [28, 29, 40].
3. Cultural Influences: Local beliefs shaped perceptions; interventions aligned with culture were better received [46, 54].
4. Stakeholder engagement: Codesign with caregivers, clinicians and others ensured usability and trust [36, 37].
5. Technological feasibility: Mobile tools showed promise but faced infrastructural and literacy challenges [10, 45].

### 3.5 Summary of Quality Assessment

The included studies were assessed for methodological quality using critical Appraisal Skills Programme (CASP) and Joanna Briggs Institute (JBI) tools. The overall quality of the studies ranged from moderate to high. Common limitations noted were small sample sizes, lack of longitudinal follow‐up and limited reporting of participant selection procedures. Both qualitative and observational studies were appraised accordingly, and most met most quality criteria. Overall moderate‐to‐high quality on CASP/JBI indicates medium confidence in findings such as the feasibility of culturally adapted telemedicine and the role of caregiver engagement. Yet small samples, single‐site designs and short follow‐up limit generalisability and prevent causal claims. The evidence is strong enough to guide practice (e.g., low‐bandwidth design, multilingual content, caregiver training) while underscoring the need for larger prospective studies. The methodological quality of the included studies is summarised in Table 3.

## 4 Discussion

This systematic review provides a comprehensive analysis of how telemedicine and cultural competency intersect to address dementia care challenges in SSA. Telemedicine offers critical opportunities to bridge gaps in care, particularly in underserved communities [40, 41]. However, structural barriers, including low digital literacy and poor infrastructure, hinder scalability [29, 55].

Cultural competency is vital to successful intervention, as traditional beliefs and stigma heavily influence care‐seeking [36]. Participatory, stakeholder‐driven approaches foster trust and practicality [37, 38], aligning with the CFIR and Freeman's stakeholder theory.

Ethical concerns, such as privacy and exclusion of non‐digital populations, must also be addressed [47]. Policymakers should integrate dementia care into primary healthcare, develop culturally tailored materials and invest in infrastructure.

### 4.1 Future Directions and Limitations

Future research should evaluate cost‐effectiveness, scalability, and long‐term outcomes of culturally adapted telemedicine interventions. Hybrid digital and non‐digital models could enhance inclusivity. Limitations of this review include heterogeneity of studies, small sample sizes and potential publication bias. Despite these, the review offers valuable insights and directions for research and policy.

## 5 Conclusion

This review underscores that effective dementia care in SSA requires interventions that are not only technologically sound but also culturally sensitive and grounded in stakeholder engagement. Telemedicine holds significant potential to enhance access to care, but its success hinges on addressing structural barriers and embedding cultural competence at every stage of design and implementation.

## Author Contributions

**Abiodun Adedeji:** conceptualisation, methodology, data curation, formal analysis, writing original draft, writing – review and editing. **Huseyin Dogan:** supervision and review. **Festus Adedoyin:** supervision and review. **Michelle Heward:** supervision and review.

## Ethics Statement

This systematic review did not require ethical approval as it is based on the analysis of previously published literature. No primary data collection was performed.

## Conflicts of Interest

The authors declare no conflicts of interest.

## Data Availability

All data underlying the findings of this study are fully available within the manuscript and its supporting information files.

## Appendix A Full Search Strategy for Scopus

The search in Scopus was conducted in March, 2024 using the following search string:

**Search String:**

(“dementia” OR “Alzheimer”) AND (“telemedicine” OR “digital health” OR “e‐health”) AND (“cultural competency” OR “cultural adaptation” OR “culturally appropriate”) AND (“Sub‐Saharan Africa” OR “Nigeria” OR “Kenya” OR “Ghana” OR “South Africa” OR “Tanzania”)

Filters applied:

- Language: English
- Publication type: Journal articles
- Timeframe: All years up to March, 2024

Similar strategies were adapted for other databases (CINAHL, Web of Science, Google Scholar) using equivalent keywords and Boolean operators.
